# Regulation of reproductive development by non-coding RNA in Arabidopsis: to flower or not to flower

**DOI:** 10.1007/s10265-012-0513-7

**Published:** 2012-07-27

**Authors:** Ayako Yamaguchi, Mitsutomo Abe

**Affiliations:** 1 Laboratory of Plant Science, Department of Biological Sciences, Graduate School of Science, The University of Tokyo, 7-3-1, Hongo, Bunkyo-ku, Tokyo, 113-0033 Japan; 2 Present Address: Graduate School of Biostudies, Kyoto University, Yoshida Konoecho, Sakyo-ku, Kyoto, 606-8501 Japan

**Keywords:** *Arabidopsis thaliana*, Flowering, Long noncoding RNA (lncRNA), MicroRNA (miRNA), Reproductive competency, Vernalization

## Abstract

Plants monitor environmental factors, such as temperature and day length, and also endogenous factors, such as their age and phytohormones, to decide when to flower. These cues are utilized to control expression levels of genes required for flowering. Thus, flowering time control is a unique model for understanding how gene activity is precisely regulated at the transcriptional level. In Arabidopsis, a remarkable number of non-coding RNA molecules have been identified by advanced sequencing technology. Recent progress in the flowering field has revealed several non-coding RNAs that play a major role in determining flowering time. Here, we introduce how two types of non-coding RNA species, microRNA (miRNA) and long noncoding RNA (lncRNA), contribute to flowering via regulation of target gene activity involved in this vital developmental transition.

## Introduction

**Fig. 1:**
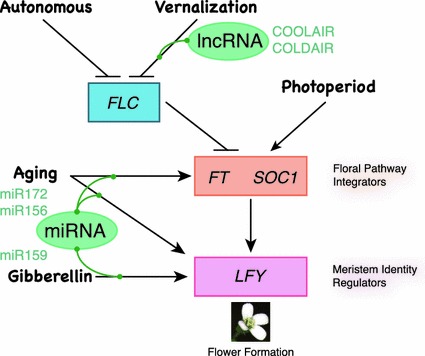
Major pathways and non-coding RNAs for flowering time control. There are five major pathways controlling flowering time in Arabidopsis; autonomous, vernalization, photoperiod, aging and gibberellin pathway. The function of the autonomous and vernalization pathways is to repress the activity of *FLOWERING LOCUS C* (*FLC*), a repressor of flowering, via chromatin modifications of the *FLC* locus. The targets of repression by *FLC* are *FLOWERING LOCUS T* (*FT*) and *SUPRESSOR OF OVEREXPRESSION OF CO1* (*SOC1*), also known as floral pathway integrators. *FT* and *SOC1* are positively regulated by the photoperiod pathway. Environmental inputs integrated by *FT* and *SOC1* hubs induce expression of meristem identity regulators to start flower formation. The gibberellin pathway mainly regulates *LEAFY* (*LFY*) expression. The recently identified aging pathway affects flowering time in two ways; first, it represses the activity of repressors of flowering, allowing plants to respond to flowering stimuli, secondly, it directly regulates the floral pathway integrators and meristem identity regulators. Two long noncoding RNAs (lncRNAs) are involved in the regulation of *FLC*. The aging pathway and gibberellin pathway accompany with the microRNA (miRNA) activity

Plants sense multiple environmental and endogenous signals to determine when to flower. For sessile plants, the ability to monitor and integrate multiple signals is essential to succeed in reproduction. Hence, a complex gene regulatory network has been constructed to enable plants to flower appropriately in time and space. Genetic and molecular analyses of flowering-time mutants in Arabidopsis has established the current model, in which five major genetically defined pathways regulate the transition from the vegetative to reproductive phase (Fig. 1; details are reviewed in Srikanth and Schmid 2011). The photoperiod pathway regulates flowering time based on day length. The vernalization pathway mediates the response to low temperature over long periods, enabling plants to sense and remember winter has come and gone. The autonomous pathway acts independently from the photoperiod and vernalization pathways, and shares a few same targets with the vernalization pathway. The gibberellin pathway defines a requirement for gibberellic acid (GA), primarily under unfavorable conditions, for flowering. The former two pathways are responsible for responding to the appropriate environmental conditions, and the latter two, reflect the endogenous status of plants.

Recently, the fifth pathway named the aging pathway, has been described by several groups (Wang et al. 2009; Wu et al. 2009; Yamaguchi et al. 2009). The importance of the endogenous status of “age” in flowering has long been recognized, since there is a reproductively incompetent stage, especially in woody plants. As plants grow, they acquire competency to respond to stimuli to produce flowers. In the case of the annual herbaceous plant Arabidopsis, there is a clear phase in which seedlings cannot respond to photoperiods (Mozley and Thomas 1995). The aging pathway is involved in the process to acquire this reproductive competency.

These five flowering pathways together constitute a complex gene-network that exhibits crosstalk, feedback-loops and redundancies. However, this complexity as a whole can be viewed as a mechanism for the precise regulation of the activity of a relatively small number of genes, which constitute regulatory “hubs”. *FLOWERING LOCUS C* (*FLC*), *FLOWERING LOCUS T* (*FT*), *SUPRESSOR OF OVEREXPRESSION OF CO1* (*SOC1*) and *LEAFY* (*LFY*) are important regulatory hubs in the control of flowering time. *FLC* expression is regulated by the vernalization pathway and the autonomous pathway, respectively. *FLC* encodes a MADS-box transcription factor and acts as a dominant repressor of flowering through its control of *FT* and *SOC1*. *FT* and *SOC1* are both targeted by the photoperiod pathway at the transcriptional level as well. The activation of *FT* is an especially crucial step in the decision to flower, since the protein encoded by *FT* is a key component of the systemic flowering signal “florigen”. After integration of environmental and endogenous inputs at the leaves via transcription of *FT*, the FT protein travels to the apical meristem where it initiates floral development (Abe et al. 2005; Wigge et al. 2005; Corbesier et al. 2007; Jaeger and Wigge 2007; Lin et al. 2007; Mathieu et al. 2007; Notaguchi et al. 2008). The final important step to confine the floral meristem at the flanks of the apical meristem is the induction of floral meristem identity regulators, such as *LFY*. The plant specific transcription factor LFY activates many other target genes to evoke the program for flower formation. LFY plays a role as a “hub” by integrating signals from the GA and the aging pathways, respectively.

Transcription of these regulatory hubs is tightly regulated at multiple levels. In general, tissue- and/or developmental stage-specific transcription factors can confer a specific expression profile in time and space. Regulatory information for multiple upstream transcription factors is often present on the same promoter for particular genes, which enables fine-tuned precise expression of the associated gene. At another level of regulation, chromatin modifications play an important role in regulating *FLC* expression (reviewed in Kim et al. 2009). Chromatin modifications are reported to regulate the levels of transcripts and/or the developmental stage specificity of expression. There are also the observations in which *FT* and *LFY* expression are affected by chromatin modifiers (Adrian et al. 2011; Kinoshita et al. 2001).

Recently, a remarkable number of non-coding RNA molecules have been identified by advanced sequencing technologies. Intriguingly, these non-coding RNAs have received attention as another level of regulation for controlling flowering time via regulating the expression of key players. In this review, two types of non-coding RNA will be introduced; microRNAs (miRNAs) and long noncoding RNAs (lncRNAs). The function of these non-coding RNAs in the control of flowering will be described. In addition, we will discuss the future challenges in this field.

## MicroRNA and flowering time control

### Biogenesis and action of miRNAs in Arabidopsis

MicroRNAs are small 21–22 nucleotide (nt) RNAs that repress specific target mRNA activity at the transcriptional and/or translational level. In the early 1990s, miRNAs were discovered from the developmental timing studies in *Caenorhabditis elegans*. The *lin*-*4* and *let*-*7* miRNAs were identified as silencers of genes important for the transition from one larval stage to another (Lee et al. 1993; Reinhart et al. 2000). Not much later, multiple miRNA families were also discovered in the plant kingdom, and shown to be involved in various developmental events including flowering (Llave et al. 2002; Park et al. 2002; Reinhart et al. 2002).

**Fig. 2:**
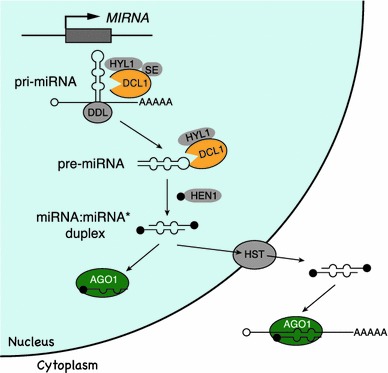
miRNA biogenesis in Arabidopsis. Expression of miRNAs begins with transcription of the miRNA locus by pol II, then pri-miRNAs are processed through multi-steps by the RNase like enzyme DCL1 to generate the miRNA:miRNA* duplex. This duplex is modified at its 3′ terminus by HEN1, which prevents it from further degradation. Only the guide strands are sorted to AGO1 at the nucleus or the cytoplasm, and this leads to transcriptional or translational silencing. Refer to the text for more details

The mechanism for miRNA biogenesis in plants has been well studied in Arabidopsis (Fig. 2; reviewed in Mallory et al. 2008; Xie et al. 2010). The first step of miRNA biogenesis is similar to the generation of mRNA. The primary miRNA (pri-miRNA) is transcribed by RNA polymerase II and has features in common with mRNA, including a 5′ cap and 3′ poly(A) tail. In addition, pri-miRNAs contain stem-loop structures that are thought to be stabilized by the nuclear RNA binding protein DAWDLE (DDL) (Yu et al. 2008). This ability to form a hairpin structure distinguishes miRNAs from other types of small RNAs.

The processing of pri-miRNAs occurs in two steps. The pri-miRNA is first cleaved into a precursor miRNA (pre-miRNA). The pre-miRNA is then cleaved into the mature miRNA:miRNA* duplex. DICER-LIKE 1 (DCL1), a homolog of the RNase III-like enzyme Dicer protein in animals, catalyzes the cleavage of both the pri-miRNA and the pre-miRNA. The first processing step to generate pre-miRNA is assisted by a dsRNA binding protein HYPONASTIC LEAVES 1 (HYL1) (Han et al. 2004; Kurihara et al. 2006; Vazquez et al. 2004) and the C2H2 zinc finger protein SERRATE (SE) (Dong et al. 2008; Kurihara et al. 2006; Lobbes et al. 2006; Yang et al. 2006). After the pre-miRNA is further processed by DCL1 and converted into the miRNA:miRNA* duplex, the 3′ ends of this miRNA:miRNA* duplex are modified by the methyltransferase HUA ENHANCER1 (HEN1). Plant HEN1 is a nuclear protein that adds methyl groups to both strands of the miRNA:miRNA* to protect it from further modifications such as 3′ uridylation and subsequent degradation (Li et al. 2005; Yang et al. 2006; Yu et al. 2005).

After modification by HEN1, the miRNA:miRNA* duplex is thought to be transported out from the nucleus to the cytoplasm by the function of the Exportin 5 homolog HASTY (HST), or through a HST independent mechanism (Park et al. 2005), then assembled into an RNA induced silencing complex (RISC) containing the ARGONAUTE (AGO) protein. When loading into RISC, one strand from the miRNA:miRNA* duplex is selected and stabilized. Recently, it was shown that cyclophilin 40, SQUINT (SQN), and the heat shock protein 90 (HSP90) genetically and biochemically interact with AGO1 to promote AGO activity for the loading and selecting process (Earley and Poethig 2011; Iki et al. 2011; Smith et al. 2009).

Finally, the selected strand guides RISC to the target mRNA which has complementary sequences to the miRNA. In plants, binding of a miRNA to its target mRNA mainly causes mRNA degradation triggered from a single endonucleolytic cleavage at the middle of the mRNA-miRNA duplex by the RNase H activity of AGO1. There are also a few examples demonstrating that miRNAs cause translational repression in plants (Yang et al. 2012), although this mechanism is relatively unclear compared to transcriptional silencing by miRNAs.

### Role of miRNAs in flowering

Crucial functions of miRNAs in plant development are supported by the fact that mutations in genes involved in the miRNA biogenesis pathway show severe developmental defects. Bohmert et al. (1998) reported that null alleles of *DCL1* and *AGO1* lead to embryonic lethal phenotypes. In the case of weak alleles for these genes, the reduction of a variety of miRNAs causes pleiotropic developmental defects in leaf morphology, flowering time, flower formation and stem cell maintenance (Kidner and Martienssen 2005; Park et al. 2002; Vaucheret et al. 2004).

**Fig. 3:**
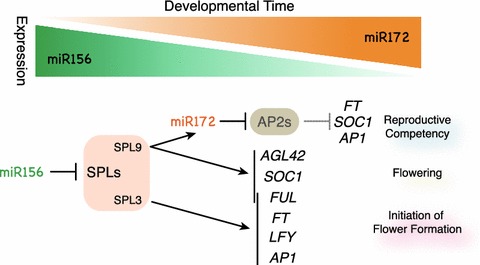
The antagonistic activity of miR156 and miR172 in the aging pathway. The nature of the aging pathway depends on the sequential activity of two miRNA families. Levels of miR156 decline as plants become older. On the other hand, miR172 levels increase as plants become older. Among the 11 *SPL* genes targeted by miR156, *SPL9* is responsible for the up-regulation of miR172b, which results in reduction of AP2-type transcription factor activity, thereby enabling plants to acquire competency to respond to proper inputs from the environment to flower. In addition to miR172b, *SPL9* can also directly up-regulate *SOC1*, *FUL* and *AGL42*, a paralog of *SOC1*. Targets of *SPL3*; *FUL*, *FT*, *LFY* and *AP1,* are also well characterized

In Arabidopsis, there are approximately 200 miRNA families encoded by almost 300 loci (estimated by miRBase Ver18; http://www.mirbase.org/). Among numerous miRNAs, only three families so far are shown to be involved in flowering time control: miR172, miR156, and miR159. In the aging pathway, miR172 and miR156 play a major role and act sequentially in the process to acquire reproductive competency (Fig. 3; reviewed in Zhu and Helliwell 2011; Huijser and Schmid 2011). Expression of the miR172 family members are progressively up-regulated as plants develop in age, while miR156 levels decline as plants become older. This temporally opposite expression pattern reflects their roles in acquiring competency to respond to stimulus for flowering and promoting flowering. On the other hand, the miR159 family plays a role in the control of flowering via the gibberellin pathway (reviewed in Terzi and Simpson 2008). The mir159 miRNAs target the GAMYB family of MYB transcription factors. This family of transcription factors regulates transcription of genes induced by GA including the floral meristem identity regulator, *LFY*. Thus, miR159 affects *LFY* expression and promotes flower formation through GAMYB activity. We will describe the action and function of miR172, miR156 and miR159 in more detail below.

### Function of miR172 in flowering time control

The Arabidopsis genome contains five loci that generate miR172 species, *MIR172a* to *MIR172e*. Based on initial analyses of miR172 in floral patterning, miR172 is one example in which miRNAs play a role in translational repression (Chen 2004). However, recent analyses show that miR172 can also degrade target mRNA (Jung et al. 2007; Wollmann et al. 2011). In Arabidopsis, the targets of miR172 include transcripts of APETALA2 (AP2)-type genes: *AP2*, *TARGET OF EAT1* (*TOE1*), *TOE2*, *TOE3*, *SCHLAFMÜTZE* (*SMZ*) and *SCHNARCHZAPFEN* (*SNZ*).

The first observation about the function of the miR172 family in flowering time control was made by Aukerman and Sakai (2003). They identified the miR172b overexpressing line named *early activation tagged*, *dominant* (*eat*-*D*) as an extremely early-flowering mutant from an activation-tagging screen. In contrast to the early-flowering phenotype of *35S:miR172*, overexpression of *TOE1*, one of the targets of miR172, causes a late-flowering phenotype. This observation suggests that TOE1 acts as a repressor of flowering. Consistent with this, *toe1* loss-of-function mutants show a slightly early-flowering phenotype, and this phenotype is enhanced by mutations in the *TOE2* gene, which is closely related to *TOE1*. However, *toe1 toe2* double mutants still flower later than miR172 overexpressors, indicating the existence of other factors that function redundantly with *TOE1* and *TOE2* to repress flowering. Recently, it was shown that *SMZ* and *SNZ* overexpressors flower later than wild type, and *toe1 toe2 smz snz* quadruple mutants flower much earlier than *toe1 toe2* double mutants, although *smz snz* double mutants do not affect flowering time significantly (Mathieu et al. 2009). Nonetheless, the fact that quadruple mutants still flower later than *35S:miR172* suggests further redundancy among the AP2-type genes. This is confirmed by the finding that AP2 functions as a floral repressor in addition to its well-documented roles in floral patterning. Hextuple mutants defective in all six AP2-type genes flower as early as miR172 overexpressors (Yant et al. 2010).

As mentioned above, AP2-type genes that are involved in flowering time control encode the putative transcriptional repressors. Consistent with this, AP2 can directly bind to the promoter region of the floral pathway integrator *SOC1* and the meristem identity regulator *APETALA1* (*AP1*) (Yant et al. 2010). Besides these targets, binding of the AP2 protein to the *TOE3* promoter was also observed by ChIP-seq analysis (Yant et al. 2010), suggesting that there might be a feedback loop between AP2-type genes, although the significance of this loop remains unknown. Another AP2-type protein SMZ directly binds to the 1.5 kb downstream region of *FT* at the leaves and seems to repress its transcription. In addition to *FT*, *SOC1* and *AP1* are also directly targeted by SMZ at the shoot meristem (Mathieu et al. 2009). Furthermore, *FT* is also proposed as a target of TOE1 and TOE2 proteins based on the observation that *FT* expression was upregulated in *toe1 toe2* double mutants (Jung et al. 2007). The spatial expression patterns of *TOE1* in the leaf vasculature also support the idea that *FT* is a target of TOE1, although direct association of TOE1 and/or TOE2 on the *FT* promoter remains to be shown.

The transcription levels of AP2-type genes except *TOE3* decline over time after germination (Aukerman and Sakai 2003; Jung et al. 2007; Mathieu et al. 2009). This temporal pattern of expression seems to be the result of an increase in miR172 activity. Recently, the age-dependent accumulation of miR172 was shown to primarily originate from transcriptional regulation by a SBP box transcription factor, which itself is regulated by the temporally controlled miRNA, miR156 (Wu et al. 2009). Transcriptional regulation of *MIR172b* by the SBP transcription factor enables plants to acquire competency to respond to flowering stimuli through repression of AP2-type gene activity.

It was originally reported that the accumulation of the mature miR172 increases with plant age, but it is not changed by photoperiod pathway mutations, such as *constans* (*co*) (Aukerman and Sakai 2003). However, day length seems to affect miR172 levels in Arabidopsis. In long-day (LD) conditions, miR172 accumulates more than in short-day (SD) conditions (Jung et al. 2007). In contrast to the results obtained from *co* mutants, miR172 levels are decreased in *gigantea* (*gi*) mutants that act in the photoperiod pathway (Jung et al. 2007). Although GI is proposed to act upstream of *CO*, an observed decrease of miR172 accumulation is independent from CO function. This fact suggests that GI can also act independently from CO to regulate flowering via miR172 regulation.

Ambient temperature, another environmental factor regulating flowering, seems to influence miR172 levels as well. Lee et al. (2010) examined miRNA levels from plants grown at 16 °C, and identified miR172 as an ambient temperature responsive miRNA. Intriguingly, a recent report showed that the Arabidopsis RNA-binding protein FCA modulates temperature signals and regulates pri-miR172 processing (Jung et al. 2012b). As it will be described in more detail below, miR172 is a major component of the aging pathway and acts downstream of miR156. However, environmental conditions can also affect miR172 accumulation probably independently from miR156. This might enable plant architecture, regulated by the miR172, plastic to ambient environment during particular developmental stages.

### Function of miR156 in flowering time control

In the Arabidopsis genome, there are 10 loci that encode the miR156 family, including the recently identified *MIR156i* and *MIR156j* (Breakfield et al. 2012). The miR156 family targets SBP box transcription factors (Klein et al. 1996). Seventeen family members in Arabidopsis were named *SQUAMOSA PROMOTER BINDING PROTEIN LIKE* *1* (*SPL1*) to *SPL16*, including two genes, *SPL13A* and *SPL13B* that encode the same protein. Among this family, 11 members have a target site for miR156. The early-flowering phenotype of the *SPL3* overexpressor was the first evidence for the involvement of *SPL* genes in flowering time control (Cardon et al. 1999). It was also noted that *SPL3* expression levels gradually increase over developmental time (Cardon et al. 1999). In addition, Schmid et al. (2003) reported that photo-induction causes upregulation of the *SPL* genes, including *SPL3*, in a *CO* and *FT*-dependent manner. However, at that time, there were no available mutations in *SPL3* or other *SPL* genes, making it difficult to evaluate their function in flowering time control.

The identification of miR156 led to the result in which miR156 overexpression reduces the level of target *SPL* genes and causes a late-flowering phenotype (Schwab et al. 2005; Wu and Poethig 2006). On the other hand, when miR156 activity is sequestered by an overexpressed mimicry sequence against miR156 targets, plants with elevated levels of *SPL* flower extremely early (Franco-Zorrilla et al. 2007). These observations clearly indicate the importance of the miR156/SPL regulatory module in flowering time control.

Accumulation of miR156 is high in the embryo and in the early seedling stage (Nodine and Bartel 2010; Wu and Poethig 2006), and declines as the plant grows (Schmid et al. 2003; Wang et al. 2009; Wu et al. 2009; Wu and Poethig 2006). So far, accumulation levels of miR156 were examined in several mutants and transgenic plants that exhibit flowering phenotypes (Wang et al. 2009). However, none of these mutations affect miR156 levels. Furthermore, the plant hormones, gibberellin and auxin, also have no effect on miR156 accumulation (Wang et al. 2009). Based on these observations, it is likely that miR156 levels are independent from many of the factors that affect flowering time, instead, miR156 levels are mostly dependent on plant age.

In addition to *SPL3*, most of the other miR156-targeted-*SPL* genes also show a gradual increase in their expression level during development (Cardon et al. 1999; Schmid et al. 2003; Wang et al. 2008, 2009; Wu et al. 2009; Wu and Poethig 2006; Yamaguchi et al. 2009). This temporal expression pattern is a reflection of miR156 activity and is important for normal development in terms of flowering time. When the regulation by miR156 is disturbed by mutations at the miR156 target sites in *SPL* genes, plants can flower earlier than normal with elevated levels of *SPL* expression (Gandikota et al. 2007; Wang et al. 2008, 2009; Wu and Poethig 2006). This early flowering phenotype is consistent with the original finding made with the *SPL3* overexpressor, which lacked the 3′ UTR containing the miR156 target site (Cardon et al. 1999).

The identification of direct targets of *SPL* genes has revealed the molecular details of how the miR156/SPL module is involved in the control of flowering time. The miR156/SPL module appears to regulate flowering time in two different ways; first, by eliminating the repressive state for flowering produced by AP2-type gene activity via regulation of miR172: second, by directly promoting floral pathway integrators and meristem identity regulators. SPL9 was demonstrated to directly bind to the regulatory region of *MIR172b* and induce its expression (Wu et al. 2009). As mentioned in the former section, miR172 represses the floral repressor AP2-type transcription factors, allowing plants to respond to the proper stimuli to induce flowering. It is intriguing that the sequential action of miRNAs is also observed in the regulation of developmental timing in *C. elegans* (Lee et al. 1993; Reinhart et al. 2000). In addition to miR172, the floral pathway integrator *SOC1* and its paralog *AGL42* were identified as direct targets of SPL9 (Wang et al. 2009). Although the role of *FUL* in flowering time has been underestimated, *FUL* is regulated by SPL9 and SPL3 and is responsible for the early-flowering phenotypes of the *SPL9* and *SPL3* overexpressors (Wang et al. 2009; Yamaguchi et al. 2009). Furthermore, SPL3 protein directly regulates the meristem identity regulators *LFY*, *AP1* and *FUL* (Yamaguchi et al. 2009). In addition, direct interaction of SPL3 on the promoter region of *FT* was shown recently (Kim et al. 2012). In this report, *FT* is proposed as a major output of the miR156/SPL3 module, which regulates the ambient temperature responsive flowering pathway.

Although the temporal accumulation pattern of miR156 is key in the aging pathway, the mechanism by which this temporal pattern originates from remains completely elusive and is an interesting challenge for future study. The decline in the miR156 accumulation levels originates at least from decreased activity at the transcriptional level, since pri-miR156a expression decreases over time (Wang et al. 2009). Interestingly, it was reported that if leaves already formed are defoliated, miR156 levels do not decrease (Yang et al. 2011). This observation indicates that leaves are the source of the signal(s) regulating miR156 accumulation. In addition, it was shown recently that ambient temperature affects the accumulation of miR156 and miR172 (Kim et al. 2012; Lee et al. 2010), suggesting that environmental factors can modulate plant developmental stages via affecting accumulation of these two miRNAs.

The temporal pattern of *SPL* accumulation might also be an important step to understanding the aging pathway and the complex interaction between age and environmental inputs. As described earlier, photo-induction can induce *SPL* gene expression dependent on *CO* or *FT* activity without affecting miR156 levels (Schmid et al. 2003; Wang et al. 2009). Based on recent reports, this induction seems to be directly regulated by SOC1 and FD (Jung et al. 2012a). The interaction between the aging pathway and the photoperiod pathway may not be simple, and needs to be further examined.

### Function of miR159 in flowering time control

The third miRNA species, miR159, plays a role in the gibberellin pathway. In Arabidopsis, the effect of GA on flowering was underestimated since the GA deficient mutant *ga1* does not show a significant flowering phenotype in LD conditions. On the other hand, when grown under SD conditions, the *ga1* mutant requires GA for flowering (Wilson et al. 1992). However, recent identification of the GA receptor, GIBBERELLIC INSENSITIVE DWARF1 (GID1), allowed a role for GA in flowering time to be reconsidered. Triple mutants of the three functionally redundant copies of the GID1 receptor flower late even under LD conditions (Griffiths et al. 2006; Willige et al. 2007). This suggests that GA acts as an important cue in flowering time control under LD conditions.

One of the molecular targets of the gibberellin pathway has long been proposed to be *LFY*. GA signals are mediated via the GAMYB binding elements on the *LFY* promoter and the application of GA elevates the promoter activity of *LFY* even in SD (Blazquez et al. 1998; Blazquez and Weigel 2000). The fact that a member of the Arabidopsis GAMYBs, MYB33, binds to the GAMYB binding motif of the *LFY* promoter in vitro and its expression can respond to an increase of endogenous levels of GA or exogenous applications of GA suggests that GAMYBs play an essential role in the GA-mediated promotion of flowering (Gocal et al. 2001).

In Arabidopsis, MIR159a, *MIR159b* and *MIR159c* comprise the miR159 family. These miRNAs mainly target the GAMYB-type transcription factors, MYB33, MYB65, and MYB101, which are potential homologues of the barley GAMYBs. A recent study has shown that *MYB33* expression is strongly repressed by miR159 in vegetative tissues (Alonso-Peral et al. 2010). Furthermore, when miR159 is overexpressed, plants flower late in SD conditions with decreased levels of *MYB33* and *LFY* (Achard et al. 2004). These observations at least suggest that miR159 is involved in flowering time control in part via the gibberellin pathway. However, the relationship between miR159 levels and GA is not as clear as expected, since miR159 levels are eliminated in GA biosynthesis mutants and exogenous applications of GA induce miR159 levels in a DELLA protein dependent manner (Achard et al. 2004). Further investigations are required to elucidate the precise interaction between miR159 and GA.

### Future challenges; what should we learn next?

As described earlier, mutations in miRNA biogenesis components cause pleiotropic developmental defects by affecting accumulation of multiple miRNA families, indicating that modification of miRNA biogenesis activity usually causes a broad impact on the entire profile of miRNA accumulation. Therefore, accumulation of a particular miRNA is likely primarily controlled through transcriptional regulation of its pri-miRNA. Although transcriptional regulation of miR172 has been studied and *SPL9* was identified as a direct upstream regulator, it remains elusive how transcription of the three miRNA families discussed here are regulated. Given their important function in determining flowering time, it is essential to understand how these miRNAs are regulated by age, photoperiod, ambient temperature, and GA, respectively. In general, the transcription of miRNAs appears to be regulated in a manner similar to the regulation of mRNAs, by *cis*-regulatory elements and *trans*-acting factors. Interestingly, genomic approaches suggest that several transcription factors, well known as a developmental regulators, target the miRNA promoters. The binding motifs of LFY, Auxin response factors (ARFs) and AtMYC2 were found more frequently in the promoters of miRNAs compared with the promoters of protein coding loci (Megraw et al. 2006). It might be useful to further validate these predictions in an experimental context.

Studies to understand the spatial pattern of miRNA activity from the transcription of their pri-miRNAs to repression of their targets are also important. Although multiple loci encode one particular miRNA family, only a few loci are responsible for accumulation of the mature miRNA; for example, *MIR172b* for the temporal accumulation of the mature miR172. We need to focus on such loci to analyze when and where their promoters are activated. In addition, the spatial pattern of mature miRNA accumulation and activity needs to be analyzed at high resolution as done in Nodine and Bartel (Nodine and Bartel 2010), given the fact that miRNAs can function as non-cell autonomous signaling molecules (Carlsbecker et al. 2010; Miyashima et al. 2011). Recently, the cell type specific miRNA expression became available for the root, revealing that miR156 is enriched in the cortex and epidermis (Breakfield et al. 2012). Accumulation of miR156 in the phloem has also been reported (Buhtz et al. 2010). It is tempting to speculate a long-range action of specific miRNAs involved in flowering time control.

Genome information is now available from a variety of species, making it possible to answer the questions regarding the evolutionary origins of miRNAs. The miR156 and miR172 families are widely conserved in angiosperms, and play a role in the regulation of developmental timing in several plants such as maize (Chuck et al. 2007). The accumulation of miR156 and miR172 could be good markers to estimate the developmental stage of different species. Such a tool might be especially useful in woody plants (Wang et al. 2011). Although the presence of miR172 has been confirmed only in angiosperms, miR156 has been identified in moss (Arazi et al. 2005), suggesting an ancient origin of the miR156/SPL regulatory module. It will be interesting to determine the ancient role of the miR156/SPL module and how this module evolved to regulate flowering time in modern-day plants.

## Long non-coding RNA and flowering

### Action of lncRNA in regulation of gene expression

LncRNAs are defined as RNA transcripts more than 200 nucleotides in length that lack protein-coding capability. These lncRNAs may be located within the nucleus or cytosol, and may or may not have a poly(A) tail. They are often transcribed from either strand of a protein-coding locus, but also arise from intergenic regions. LncRNAs are the least well-understood among various RNA species and have been called the “dark matter” of the genome. However, recent studies with advanced sequencing technology illuminate the importance of lncRNAs in transcriptional control (reviewed in Nagano and Fraser 2011; Wang and Chang 2011). Although our knowledge is still incomplete, lncRNAs appear to influence gene expression mainly in two ways, through direct effects on transcription and recruitment of chromatin modifiers. These two prototypes are not mutually exclusive.

Many examples of direct effects of transcription have come from studies in yeast. Transcription of the lncRNA *SRG1* through the promoter of the adjacent *SER3* gene interferes with the transcription of *SER3* (Martens et al. 2004). Antisense transcripts originated from adjacent promoters can also function as enhancers of sense transcription (Camblong et al. 2007). A study of glucose starvation in yeast proposed that transcription of lncRNAs elevates the accessibility of protein-coding genes to RNA polymerases (Hirota et al. 2008). Examples in which lncRNAs directly affect transcription are also found in mammals. LncRNAs transcribed from the upstream of the human dihydrofolate reductase *DHFR* gene forms a lncRNA-DNA complex with promoter sequences and directly interacts with the general transcription factor IIB (TFIIB), which inhibits assembly of the preinitiation complex at the promoter (Martianov et al. 2007).

**Fig. 4:**
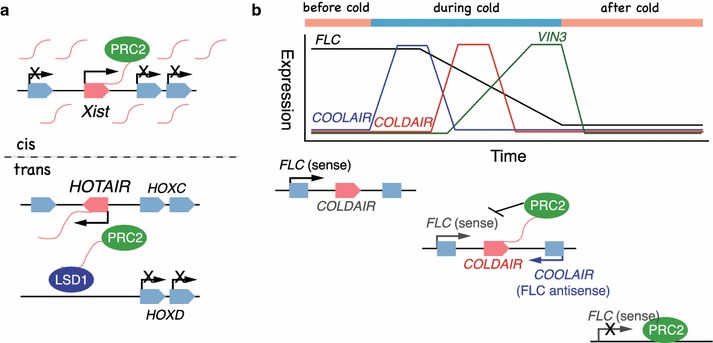
Regulation of gene expression by lncRNA. **a** Recruiting chromatin modifiers by lncRNAs. *Xist* RNA is expressed from the inactive X and establishes a nuclear domain for gene silencing via directly or indirectly recruiting the histone modifier complex, the Polycomb repressor complex 2 (PRC2) (above). *HOTAIR* RNA is transcribed from the *HOXC* cluster and interacts with PRC2, which results in the methylation and silencing of several genes in the *HOXD* cluster in *trans* (below). **b** Regulation of *FLC* by lncRNA. Before cold exposure, *FLC* is actively transcribed, at the onset of cold temperature, the *FLC* antisense transcript *COOLAIR,* accumulates. Next, COLDAIR, another lncRNA from the first intron of *FLC*, is transcribed, and represses *FLC* sense transcripts via recruiting the PRC2 complex. Following *COLDAIR* accumulation, *VIN3* expression is gradually increased depending on the length of cold exposure. Once back to normal temperature, *VIN3* levels return to the same level before the cold. This transient expression of *VIN3* is also a key to the initial repression of *FLC*. The repression of *FLC* sense transcripts is maintained by activity of PRC2 after returning to warm temperature

LncRNAs can also cause epigenetic silencing by acting as scaffolds for the recruitment of chromatin modifiers. A well studied example is the mammalian lncRNA, *Xist* RNA, that is crucial for X chromosome inactivation (Fig. 4a, above). *Xist* RNA associates with the X chromosome that is inactivated. This RNA “coat” is the first model in which lncRNAs act in epigenetic silencing. Initiation of *Xist* transcription seems to be dependent on another lncRNA, RepA RNA, which arises from the 5′ end of *Xist* (Zhao et al. 2008). RepA recruits Polycomb repressing complex 2 (PRC2) and causes trimethylation of H3K27 on the X chromosome, resulting in inactivation of the X chromosome. LncRNAs can also guide chromatin modifiers to distantly located genes (in *trans*). For example, the *HOTAIR* lncRNA, which is expressed from an intergenic region of the *HoxC* cluster, is involved in the silencing of the *HoxD* cluster located on a different chromosome (Fig. 4a, below). Although the guidance mechanism is not completely understood yet, it has been shown that *HOTAIR* can bind to the PRC2 complex and the LSD1 complex (Tsai et al. 2010). The function of lncRNAs as scaffolds for histone modifiers might be an important component of epigenetic maintenance of cell identity across cell divisions.

### Action of lncRNA for epigenetic regulation on FLC

Recently, a similar function for lncRNAs in chromatin modification is also found in the regulation of *FLC* expression, one of the key steps in Arabidopsis flowering time control (Fig. 1; reviewed in De Lucia and Dean 2011; Kim and Sung 2012a, b). High levels of *FLC* transcripts confer the vernalization requirement to many natural variants of Arabidopsis from northern Europe. Prolonged exposure of cold in winter reduces *FLC* expression, and this repressive state is maintained after returning to warmer temperatures in the spring. Dissecting the mechanisms underlying the transcriptional regulation of *FLC* has shed light on the basic principles of epigenetic silencing in plants, and now gives insight into the function of lncRNAs.

Chromatin modifications at the *FLC* locus before vernalization are characteristic of actively transcribed chromatin, consistent with the high level of *FLC* expression before cold treatment. High levels of H3K4 trimethylation are observed before cold. Complex proteins associated with the Set1 (COMPASS) complex containing H3K4 methyltransferase is responsible for adding this “active” mark on *FLC* chromatin, and this mark is recognized by the RNA polymerase II-associated factor 1 (PAF1) complex for active transcription. During cold, a dramatic change in chromatin status from “active” to “repressive” occurs at the *FLC* locus, accompanied by an increase in H3K9 and H3K27 methylation. Three key players act in this process: *VERNALIZATION INSENSITIVE 3* (*VIN3*), *VERNALIZATION 1* (*VRN1*) and *VRN2* (Gendall et al. 2001; Levy et al. 2002; Sung and Amasino 2004). *VIN3* encodes a PHD domain protein and its expression is gradually induced by exposure to cold (Fig. 4b above). Reduction of *FLC* does not occur in *vin3* mutants, suggesting the essential role of VIN3 for establishing the repressive status of the *FLC* locus. Supporting this idea, the VIN3 protein is reported to interact with the PRC2 protein (Wood et al. 2006), although how this interaction affects histone modifications needs to be addressed. The VRN1 and VRN2 protein maintain the H3K9 and H3K27 methylation caused by VIN3 at the *FLC* locus. The VRN2 protein shows similarity to the suppressor of zeste (Su(z)12) protein, which is a component of the PRC2 complex. *VRN1* encodes a plant specific DNA binding protein and acts in an alternative PRC1 like complex in plants.

LncRNAs add another regulatory layer in the control of *FLC* transcription. Recently, the rapid accumulation of *FLC* antisense transcripts were found as one of the earliest events in the vernalization process (Swiezewski et al. 2009). This antisense transcript named as *COLD INDUCED LONG ANTISENSE INTRAGENIC RNA* (*COOLAIR*) accumulates earlier than *VIN3* induction (Fig. 4b, above; Liu et al. 2010). The *COOLAIR* promoter is cold inducible and can induce cold-dependent silencing of the heterologous reporter when added to the end of the reporter. From these findings, *COOLAIR* is proposed to silence the sense transcript of *FLC* by promoting PRC2 recruitment. Although the function of *COOLAIR* in the vernalization process should be validated carefully since a contradictory result has also been reported (Helliwell et al. 2011), there is an intriguing characteristic of the *COOLAIR* transcript. It has two polyadenylation sites at the distal and proximal region respectively. COOLAIR polyadenylated at the distal 3′ end is associated with high expression of *FLC*. Alternatively, polyadenylation at the proximal 3′ end is associated with low expression of *FLC*. Two autonomous pathway genes, *FCA* and *FPA,* encoding the RNA binding proteins, promote 3′ processing at the proximal polyadenylation site of the *COOLAIR* transcript, resulting in H3K4 demethylation of *FLC* (Hornyik et al. 2010; Liu et al. 2010). This causes down-regulation of *FLC* transcription and promotes flowering. Although the importance of 3′ processing in lncRNA function has not been described in other organisms yet, it is tempting to speculate it as a common mechanism.

More recently, the second lncRNA named *COLD ASSISTED INTRONIC NONCODING RNA* (*COLDAIR*) was identified as a regulator for epigenetic silencing of *FLC* (Fig. 4b; Heo and Sung 2011). *COLDAIR* is a 1.1 kb transcript from the first intron of *FLC* in the sense direction with a 5′ capped end but without a 3′ poly(A) tail, which is similar to transcripts produced by RNA polymerase V (PolV). However, mutations in PolIV or PolV subunits does not affect *COLDAIR* accumulation induced by cold. On the other hand, PolII occupancy is transiently increased by cold, suggesting that PolII is responsible for the transcription of *COLDAIR*. Intriguingly, CURLY LEAF (CLF), the H3K27 trimethyltransferase in the PRC2 complex of plants can bind to *COLDAIR* lncRNA. The authors also showed that reduced *COLDAIR* transcripts by RNAi compromises the vernalization response. These observations point to a similarity in the action of *COLDAIR* lncRNA and *HOTAIR* lncRNA as scaffolds for histone modifiers. It will be interesting to further explore the role of *COLDAIR* in recruitment and maintenance of the PRC2 association with the *FLC* locus.

### Future challenge; how is transcription of lncRNAs regulated?

Two lncRNAs identified from the studies of epigenetic regulation of *FLC* are both clearly induced by exposure to cold, marking the very beginning of the vernalization process. This induction is even earlier than *VIN3*, which is thought to be a key determinant of long exposure to cold (Fig. 4b). Although lncRNAs have long been thought of as transcriptional “noise”, after advances in RNA sequencing technology, it is now clear that many lncRNAs show cell-type specific expression and response to diverse stimuli, suggesting lncRNAs function as molecular signals. This prompts us to consider the transcriptional regulation of lncRNAs. Interestingly, in the case of *Xist* RNA, an antisense lncRNA named *Tsix* has been shown to repress *Xist* RNA transcription in *cis*, suggesting the presence of another layer of regulation (Tsai et al. 2010). Studying the transcriptional regulation of the lncRNAs *COOLAIR* and *COLDAIR*, is a promising research endeavor to acquire further information on the regulatory mechanism and molecular signals that are initiated during cold exposure. In addition, it will provide us clues to understand the sensing of cold temperatures, which is currently the least described step in the vernalization process.

## Concluding remarks

The advancement of sequencing technology has expanded the list of non-coding RNA species, while studies of flowering time have significantly contributed to our understanding of the function and action of miRNAs and lncRNAs as described in this review. With regard to the function of lncRNAs in plants, there are few reports except for the studies of the vernalization process. The advantage of using non-coding RNA to regulate flowering time might be related to the quantitative nature of flowering time. Using RNA as a medium may facilitate quantitative control of the key transcripts in the determination of flowering time. It might be useful to perform a regulatory process quickly without protein translation. Further studies of flowering time control promise to bring us further general insights into the regulatory mechanisms of gene expression.
